# Crystal structure of (1S*,2R*)-7-benz­yloxy-2-methyl-3-tosyl-2,3,4,5-tetra­hydro-1*H*-3-benz­azepin-1-ol: elucidation of the relative configuration of potent allosteric GluN2B selective NMDA receptor antagonists

**DOI:** 10.1107/S2056989016005855

**Published:** 2016-04-15

**Authors:** Bastian Tewes, Bastian Frehland, Roland Fröhlich, Bernhard Wünsch

**Affiliations:** a Institut für Pharmazeutische und Medizinische Chemie der Universität Münster, Corrensstrasse 48, D-48149 Münster, Germany; b Organisch-chemisches Institut der Westfälischen Wilhelms-Universität Münster, Corrensstr. 40, D-48149-Münster, Germany; c Cells-in-Motion Cluster of Excellence (EXC 1003 – CiM), Universität Münster, Germany

**Keywords:** crystal structure, NMDA receptor antagonists, GluN2B antagonists, ifenprodil analogs, tetra­hydro-3-benzazepines, relative configuration, conformational restriction, hydrogen bonding

## Abstract

In the title compound, C_25_H_27_NO_4_S, which crystallized as a racemate, the relative configuration of the adjacent OH and CH_3_ groups on the azepine ring is *trans*. The seven-membered azepin ring has a chair-like conformation. The planar aromatic rings of the benzyl and tosyl­ate moiety are inclined to the planar 3-benzazepine ring by 78.39 (15) and 77.03 (14)°, respectively, and to each another by 13.82 (15)°. In the crystal, mol­ecules are linked *via* O—H⋯O and C—H⋯O hydrogen bonds, forming double-stranded chains along the *a-*axis direction. The chains are linked *via* C—H⋯π inter­actions, forming a three-dimensional architecture.

## Abstract 2

Tetra­hydro-3-benzazepines with a hy­droxy group in the 1-position and a methyl group in the 2-position were designed as conformationally restricted ifenprodil analogues. The enanti­omerically pure 3-benzazepine (*S*,*R*)-**4** representing a constitutional isomer of ifenprodil shows high affinity towards the ifenprodil binding site (Ki = 26 n*M*) and high antagonistic activity at the NMDA receptor (IC50 = 9.0 n*M*). The crystal structure analysis of the inter­mediate sulfonamide (*S*,*R*)-2 was performed in order to assign unequivocally the relative configuration of the methyl and hy­droxy groups.

## Chemical context   

Inhibition of overactive *N*-methyl-d-aspartate (NMDA) receptors represents a promising strategy for the treatment of acute (*e.g.* stroke, epilepsy, traumatic brain injury) and chronic neuronal disorders (*e.g.* neuropathic pain, depression, Alzheimer’s and Parkinson’s disease) (Bräuner-Osborne *et al.*, 2000 ▸; Kew & Kemp, 2005 ▸; Paoletti *et al.*, 2013 ▸; Wu & Zhou, 2009 ▸). The NMDA receptor consists of four proteins (hetero­tetra­mer), which form a cation channel allowing the penetration of Ca^2+^ and Na^+^ ions into the neuron (Furukawa *et al.*, 2005 ▸). In particular, NMDA receptors containing the GluN2B subunit are an attractive target for the development of innovative drugs, since the expression of the GluN2B subunit is limited to only a few regions of the central nervous system, including cortex, striatum and hippocampus (Borza & Domány, 2006 ▸; Layton *et al.*, 2006 ▸; Mony *et al.*, 2009 ▸). Moreover, the GluN2B subunit can be addressed selectively by ligands inter­acting with the so-called ifenprodil binding site, which is formed at the inter­face between GluN2B and GluN1 subunits (Karakas *et al.*, 2011 ▸; Paoletti *et al.*, 2013 ▸).

**Figure 1:**
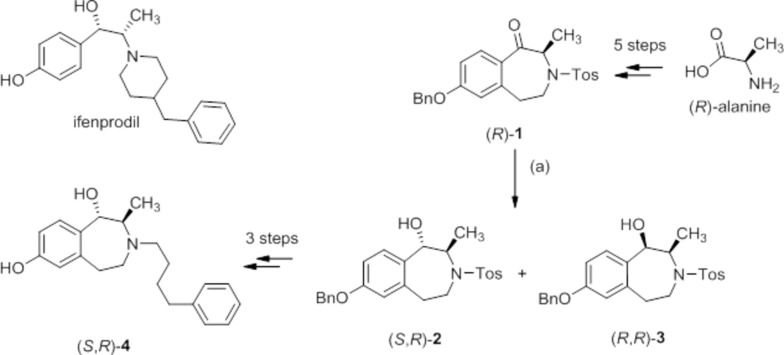
Synthesis of GluN2B antagonists including the lead compound ifenprodil and the target compound (*S*,*R*)-**4**. Reagents and reaction conditions: (*a*) NaBH_4_, CH_3_OH, (*S*,*R*)-**2** 50%, (*R*,*R*)-**3** 23%.

The 2-piperidino-1-phenyl­propan-1-ol derivative ifenprodil (Paoletti *et al.*, 2013 ▸; Williams, 2001 ▸) (Fig. 1 ▸) represents the first ligand inter­acting with this binding site at the NMDA receptor. As a result of its poor selectivity and low bioavailability, ifenprodil has not been developed as a drug for clinical use. In order to improve the selectivity and metabolic stability, the flexible β-amino­alcohol substructure of ifenprodil has been incorporated into a rigid tetra­hydro-3-benzazepine ring (Tewes *et al.*, 2010*a* ▸,*b* ▸; Schepmann *et al.*, 2010 ▸; Falck *et al.*, 2014 ▸).

## Elucidation of the relative configuration   

For the synthesis of 3-benzazepine analogs of ifenprodil, we developed a chiral pool synthesis starting with (*R*)-alanine. In a five step synthesis (Fig. 1 ▸), the central inter­mediate ketone (*R*)-**1** was prepared from (*R*)-alanine (Tewes *et al.*, 2015 ▸). 

The reduction of the ketone (*R*)-**1** with NaBH_4_ led to the diastereomeric alcohols (*S*,*R*)-**2** and (*R*,*R*)-**3**, which were further transformed into potent GluN2B antagonists by reductive removal of the tosyl group, alkyl­ation with 1-chloro-4-phenyl­butane and finally, hydrogeno­lytic cleavage of the benzyl ether. For example, the phenol (*S*,*R*)-**4** displays very high affinity towards the ifenprodil binding site of the NMDA receptor (*K*_i_ = 26 n*M*) and, moreover, (*S*,*R*)-**4** is able to reduce the glutamate- and glycine-induced cytotoxicity with an IC_50_ value of 9.0 n*M* (Tewes *et al.*, 2015 ▸).
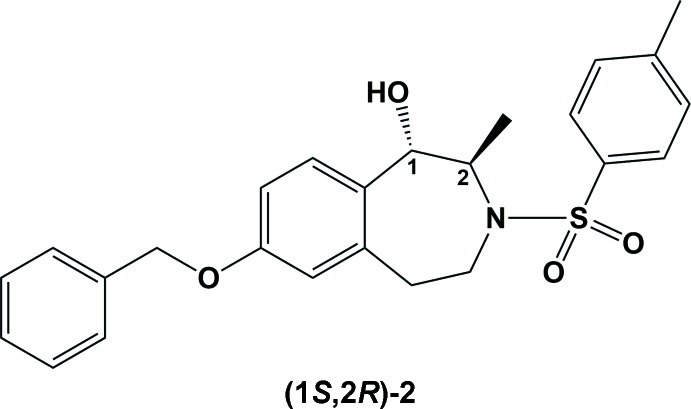


The diastereomeric alcohols (*S*,*R*)-**2** and (*R*,*R*)-**3** were separated by flash column chromatography and isolated in 50% and 23% yield, respectively. However, as a result of flexibility of the seven-membered tetra­hydro-3-benzazepine ring, it was not possible to assign the relative configuration of the methyl and hy­droxy moiety. Therefore, the main diastereomer (1*S*,2*R*)-**2** was crystallized and we report herein on its crystal structure.

## Structural commentary   

**Figure 2:**
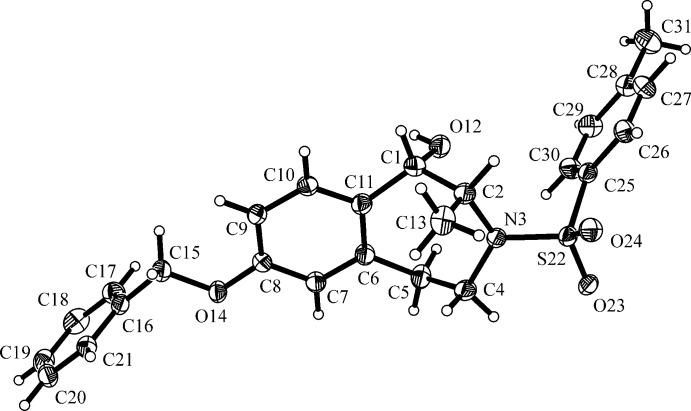
The mol­ecular structure of the title compound (1*S*,2*R*)-**2** with atom labelling. Displacement ellipsoids are drawn at the 30% probability level.

The mol­ecular structure of the title compound (1*S*,2*R*)-**2** is illustrated in Fig. 2 ▸. Since the starting material was not enanti­omerically pure, the compound crystallized as a racemate. However, the relative *trans*-configuration of the OH and CH_3_ groups in the 1- and 2-positions on the azepine ring is clearly shown, leading to a *trans*-configuration for compound (S*,R*)-**2**. The CH_3_ and the OH groups adopt an axial orientation in the seven-membered azepine ring which has a chair conformation. The phenyl group of the benzyl moiety (C16–C21) and the phenyl group of the tosyl­ate moiety (C25–C30) are inclined to the benzene ring of the 3-benzazepine ring (C6–C11) by 78.39 (15) and 77.03 (14)°, respectively, and to each another by 13.82 (15)°. In the azepine ring, the bonds between the N atom, N3, and its adjacent C atoms, C2 and C4 [1.483 (3) and 1.480 (3) Å, respectively] are naturally shorter than the corresponding C—C bonds [1.509 (4)–1.519 (4) Å]. The exocyclic N3—S22 bond is considerably longer at 1.622 (2) Å. The bond angles within the azepine ring are close to the tetra­hedral angle [106.2 (2)–116.3 (2) °]. Fig. 2 ▸ also shows the tetra­hedral geometry around the S atom, S22, of the sulfon-amide.

## Supra­molecular features   

**Figure 3:**
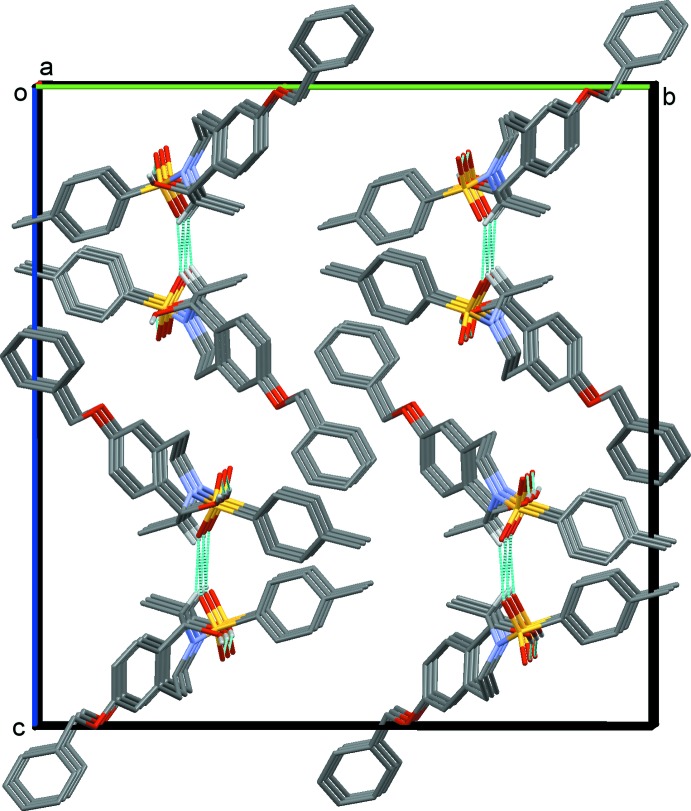
A view along the *a* axis of the crystal packing of the title compound (1*S*,2*R*)-**2**. The hydrogen bonds are shown as dashed lines (see Table 1 ▸); for clarity, H atoms not involved in these inter­actions are omitted.

In the crystal, mol­ecules are linked *via* O—H⋯O and C—H⋯O hydrogen bonds, forming double-stranded chains along the *a-*axis direction (Table 1 ▸ and Fig. 3 ▸). The chains are linked *via* C—H⋯π inter­actions (Table 1 ▸), forming a three-dimensional architecture.

## Synthesis and crystallization   

**(1** ***S*** **,2** ***R*** **)-7-Benz­yloxy-2-methyl-3-(4-tos­yl)-2,3,4,5-tetra­hydro-1** ***H*** **-3-benzazepin-1-ol [(** ***S,R*** **)-2] and (1** ***R*** **,2** ***R*** **)-7-benz­yloxy-2-methyl-3-(4-tos­yl)-2,3,4,5-tetra­hydro-1** ***H*** **-3-benzazepin-1-ol [(** ***R,R*** **)-3]**

Details of the synthesis of the title compound are illustrated in Fig. 1 ▸.

As described for the synthesis of (*R*,*S*)-**2** and (*S,S*)-**3 (**Tewes *et al.* (2015 ▸), the ketone (*R*)-**1** (5.20 g, 12.0 mmol) was reduced with NaBH_4_ (909 mg, 23.9 mmol) in CH_3_OH (125 ml).

(*S*,*R*)-**2** (*R*_f_ = 0.29): Colourless solid, m.p. 417 K, yield 2.60 g (50%). Purity (HPLC): 98.1%, *t*_R_ = 22.6 min. [α]_D_ = +1.20 (*c* = 0.91, CH_3_OH, 2.1% ee). Spectroscopic data are given in Tewes *et al.* (2015 ▸).

(*R*,*R*)-**3** (*R*_f_ = 0.44): Colourless solid, m.p. 425 K, yield 1.20 g (23%). Purity (HPLC): 95.6%, *t*_R_ = 22.2 min. [α]_D_ = +1.89 (*c* = 0.98, CH_3_OH, 8.5% ee). Spectroscopic data are given in Tewes *et al.* (2015 ▸).

Crystals of the title compound, suitable for X-ray diffraction analysis, were obtained by recrystallization from EtOAc.

## Refinement details   

Crystal data, data collection and structure refinement details are summarized in Table 2 ▸. The OH and C-bound H atoms were included in calculated positions and treated as riding atoms: O—H = 0.83 Å, C—H = 0.94–0.99 Å with *U*_iso_(H) = 1.5*U*_eq_(O or C-meth­yl) and 1.2*U*_eq_(C) for other H atoms.

## Supplementary Material

Crystal structure: contains datablock(s) I, global. DOI: 10.1107/S2056989016005855/su5286sup1.cif

Structure factors: contains datablock(s) I. DOI: 10.1107/S2056989016005855/su5286Isup2.hkl

Supporting information file. DOI: 10.1107/S2056989016005855/su5286Isup3.cml

CCDC reference: 1472947

Additional supporting information:  crystallographic information; 3D view; checkCIF report

## Figures and Tables

**Table 1:** Hydrogen-bond geometry (Å, °) *Cg*1, *Cg*2 and *Cg*3 are the centroids of rings C6–C11, C16–C21 and C25–C30, respectively.

*D*—H⋯*A*	*D*—H	H⋯*A*	*D*⋯*A*	*D*—H⋯*A*
O12—H12⋯O23^i^	0.83	2.22	3.034 (3)	169
C2—H2⋯O24^ii^	0.99	2.52	3.265 (3)	132
C18—H18⋯*Cg*3^iii^	0.94	2.89	3.738 (4)	150
C20—H20⋯*Cg*1^iv^	0.94	2.83	3.631 (3)	144
C29—H29⋯*Cg*2^v^	0.94	2.76	3.545 (3)	142

Symmetry codes: (i) 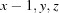; (ii) 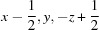; (iii) 
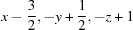
; (iv) 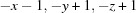; (v) 
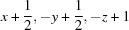
.

**Table 2:** Experimental details

Crystal data	
Chemical formula	C_25_H_27_NO_4_S
*M*_r_	437.54
Crystal system, space group	Orthorhombic, *P* *b* *c* *a*
Temperature (K)	223
*a*, *b*, *c* (Å)	7.5071 (2), 23.6113 (8), 24.5180 (8)
*V* (Å^3^)	4345.9 (2)
*Z*	8
Radiation type	Cu *K*α
μ (mm^−1^)	1.59
Crystal size (mm)	0.25 × 0.15 × 0.08

Data collection	
Diffractometer	Nonius KappaCCD APEXII
Absorption correction	Multi-scan (*DENZO*; Otwinowski *et al.*, 2003 ▸)
*T*_min_, *T*_max_	0.692, 0.884
No. of measured, independent and observed [*I* > 2σ(*I*)] reflections	40664, 3874, 3543
*R*_int_	0.064
(sin θ/λ)_max_ (Å^−1^)	0.600

Refinement	
*R*[*F* ^2^ > 2σ(*F* ^2^)], *wR*(*F* ^2^), *S*	0.058, 0.151, 1.10
No. of reflections	3874
No. of parameters	283
H-atom treatment	H-atom parameters constrained
Δρ_max_, Δρ_min_ (e Å^−3^)	0.64, −0.27

Computer programs: *COLLECT* (Nonius, 1998 ▸), *DENZO*–SMN (Otwinowski & Minor, 1997 ▸), *SHELXS97*, *SHELXL97* and *XP* in *SHELXTL* (Sheldrick, 2008 ▸), *Mercury* (Macrae *et al.*, 2008 ▸) and *PLATON* (Spek, 2009 ▸).

## supplementary crystallographic information

### Crystal data

**Table:**

C_25_H_27_NO_4_S	*F*(000) = 1856
*M**_r_* = 437.54	*D*_x_ = 1.337 Mg m^−^^3^
Orthorhombic, *P**b**c**a*	Cu *K*α radiation, λ = 1.54178 Å
Hall symbol: -P 2ac 2ab	Cell parameters from 5365 reflections
*a* = 7.5071 (2) Å	θ = 0.9–68.3°
*b* = 23.6113 (8) Å	µ = 1.59 mm^−^^1^
*c* = 24.5180 (8) Å	*T* = 223 K
*V* = 4345.9 (2) Å^3^	Plate, colourless
*Z* = 8	0.25 × 0.15 × 0.08 mm

### Data collection

**Table:**

Nonius KappaCCD APEXII diffractometer	3874 independent reflections
Radiation source: fine-focus sealed tube	3543 reflections with *I* > 2σ(*I*)
Graphite monochromator	*R*_int_ = 0.064
ω and φ scans	θ_max_ = 67.7°, θ_min_ = 4.2°
Absorption correction: multi-scan (*DENZO*; Otwinowski *et al.*, 2003)	*h* = 0→9
*T*_min_ = 0.692, *T*_max_ = 0.884	*k* = 0→27
40664 measured reflections	*l* = 0→29

### Refinement

**Table:**

Refinement on *F*^2^	Primary atom site location: structure-invariant direct methods
Least-squares matrix: full	Secondary atom site location: difference Fourier map
*R*[*F*^2^ > 2σ(*F*^2^)] = 0.058	Hydrogen site location: inferred from neighbouring sites
*wR*(*F*^2^) = 0.151	H-atom parameters constrained
*S* = 1.10	*w* = 1/[σ^2^(*F*_o_^2^) + (0.0619*P*)^2^ + 5.5349*P*] where *P* = (*F*_o_^2^ + 2*F*_c_^2^)/3
3874 reflections	(Δ/σ)_max_ = 0.001
283 parameters	Δρ_max_ = 0.64 e Å^−^^3^
0 restraints	Δρ_min_ = −0.27 e Å^−^^3^

### Special details

**Table:**

Geometry. All esds (except the esd in the dihedral angle between two l.s. planes) are estimated using the full covariance matrix. The cell esds are taken into account individually in the estimation of esds in distances, angles and torsion angles; correlations between esds in cell parameters are only used when they are defined by crystal symmetry. An approximate (isotropic) treatment of cell esds is used for estimating esds involving l.s. planes.
Refinement. Refinement of F^2^ against ALL reflections. The weighted R-factor wR and goodness of fit S are based on F^2^, conventional R-factors R are based on F, with F set to zero for negative F^2^. The threshold expression of F^2^ > 2sigma(F^2^) is used only for calculating R-factors(gt) etc. and is not relevant to the choice of reflections for refinement. R-factors based on F^2^ are statistically about twice as large as those based on F, and R- factors based on ALL data will be even larger.

### Fractional atomic coordinates and isotropic or equivalent isotropic displacement parameters (Å^2^)

**Table:**

	*x*	*y*	*z*	*U*_iso_*/*U*_eq_	
C1	−0.2632 (4)	0.25588 (13)	0.33843 (12)	0.0433 (7)	
H1	−0.3327	0.2614	0.3045	0.052*	
C2	−0.0688 (4)	0.26290 (13)	0.32342 (11)	0.0444 (7)	
H2	−0.0462	0.2383	0.2914	0.053*	
N3	0.0518 (3)	0.24359 (10)	0.36757 (9)	0.0382 (5)	
C4	0.0510 (4)	0.27343 (13)	0.42068 (11)	0.0448 (7)	
H4A	0.0656	0.3141	0.4142	0.054*	
H4B	0.1530	0.2605	0.4423	0.054*	
C5	−0.1175 (4)	0.26389 (14)	0.45294 (11)	0.0480 (7)	
H5A	−0.0934	0.2721	0.4914	0.058*	
H5B	−0.1504	0.2238	0.4502	0.058*	
C6	−0.2745 (4)	0.29959 (12)	0.43450 (11)	0.0408 (6)	
C7	−0.3588 (4)	0.33478 (12)	0.47111 (11)	0.0403 (6)	
H7	−0.3162	0.3369	0.5071	0.048*	
C8	−0.5061 (4)	0.36736 (11)	0.45600 (10)	0.0360 (6)	
C9	−0.5624 (4)	0.36714 (11)	0.40220 (11)	0.0384 (6)	
H9	−0.6574	0.3902	0.3909	0.046*	
C10	−0.4758 (4)	0.33218 (12)	0.36548 (11)	0.0415 (7)	
H10	−0.5136	0.3322	0.3289	0.050*	
C11	−0.3365 (4)	0.29737 (12)	0.38014 (11)	0.0411 (6)	
O12	−0.2846 (3)	0.19835 (9)	0.35386 (10)	0.0508 (6)	
H12	−0.3873	0.1933	0.3655	0.076*	
C13	−0.0263 (5)	0.32466 (13)	0.30574 (13)	0.0515 (8)	
H13A	−0.0412	0.3498	0.3367	0.077*	
H13B	−0.1067	0.3359	0.2767	0.077*	
H13C	0.0956	0.3268	0.2928	0.077*	
O14	−0.5837 (3)	0.39821 (8)	0.49686 (7)	0.0425 (5)	
C15	−0.7379 (4)	0.43106 (13)	0.48158 (12)	0.0459 (7)	
H15A	−0.8273	0.4065	0.4646	0.055*	
H15B	−0.7039	0.4603	0.4551	0.055*	
C16	−0.8140 (4)	0.45824 (12)	0.53175 (11)	0.0393 (6)	
C17	−0.9211 (4)	0.42782 (14)	0.56697 (13)	0.0520 (8)	
H17	−0.9428	0.3892	0.5605	0.062*	
C18	−0.9964 (5)	0.45419 (17)	0.61180 (14)	0.0611 (9)	
H18	−1.0692	0.4334	0.6357	0.073*	
C19	−0.9651 (5)	0.51087 (16)	0.62151 (13)	0.0582 (9)	
H19	−1.0168	0.5287	0.6519	0.070*	
C20	−0.8587 (4)	0.54114 (13)	0.58683 (12)	0.0499 (8)	
H20	−0.8368	0.5797	0.5936	0.060*	
C21	−0.7835 (4)	0.51513 (12)	0.54197 (12)	0.0424 (7)	
H21	−0.7110	0.5362	0.5182	0.051*	
S22	0.22307 (9)	0.20715 (3)	0.34775 (3)	0.0364 (2)	
O23	0.3341 (2)	0.19811 (9)	0.39487 (8)	0.0422 (5)	
O24	0.3039 (3)	0.23240 (9)	0.30048 (8)	0.0447 (5)	
C26	0.1770 (4)	0.12036 (12)	0.27553 (12)	0.0431 (7)	
H26	0.2349	0.1434	0.2498	0.052*	
C27	0.1279 (4)	0.06527 (13)	0.26233 (12)	0.0483 (7)	
H27	0.1544	0.0511	0.2274	0.058*	
C28	0.0412 (4)	0.03100 (13)	0.29945 (12)	0.0476 (7)	
C31	−0.0091 (6)	−0.02894 (14)	0.28515 (16)	0.0657 (10)	
H31A	−0.0590	−0.0299	0.2487	0.099*	
H31B	−0.0968	−0.0427	0.3110	0.099*	
H31C	0.0960	−0.0528	0.2866	0.099*	
C29	0.0012 (4)	0.05326 (14)	0.35077 (12)	0.0512 (8)	
H29	−0.0591	0.0306	0.3763	0.061*	
C30	0.0483 (4)	0.10756 (14)	0.36461 (12)	0.0467 (7)	
H30	0.0193	0.1222	0.3992	0.056*	
C25	0.1392 (3)	0.14065 (12)	0.32714 (11)	0.0373 (6)	

### Atomic displacement parameters (Å^2^)

**Table:**

	*U*^11^	*U*^22^	*U*^33^	*U*^12^	*U*^13^	*U*^23^
C1	0.0446 (16)	0.0458 (16)	0.0396 (14)	0.0026 (13)	−0.0051 (13)	−0.0044 (12)
C2	0.0442 (16)	0.0598 (18)	0.0294 (13)	0.0093 (14)	−0.0050 (12)	−0.0057 (12)
N3	0.0338 (11)	0.0522 (14)	0.0287 (11)	0.0052 (10)	−0.0015 (9)	−0.0057 (10)
C4	0.0424 (16)	0.0570 (18)	0.0349 (14)	0.0026 (14)	−0.0042 (12)	−0.0040 (13)
C5	0.0465 (17)	0.065 (2)	0.0320 (14)	0.0151 (15)	0.0018 (13)	0.0027 (13)
C6	0.0420 (15)	0.0484 (16)	0.0322 (14)	0.0081 (13)	−0.0002 (12)	−0.0002 (12)
C7	0.0445 (16)	0.0467 (15)	0.0295 (13)	0.0076 (13)	−0.0026 (12)	0.0003 (11)
C8	0.0392 (14)	0.0344 (13)	0.0345 (13)	0.0005 (12)	0.0011 (11)	−0.0008 (10)
C9	0.0397 (15)	0.0369 (14)	0.0387 (14)	0.0057 (12)	−0.0057 (12)	0.0002 (11)
C10	0.0465 (16)	0.0453 (16)	0.0328 (13)	0.0080 (13)	−0.0084 (12)	−0.0028 (11)
C11	0.0399 (15)	0.0465 (16)	0.0368 (14)	0.0039 (13)	−0.0019 (12)	−0.0005 (12)
O12	0.0405 (12)	0.0410 (11)	0.0709 (15)	−0.0013 (9)	−0.0018 (11)	−0.0050 (10)
C13	0.0531 (18)	0.0531 (18)	0.0483 (17)	0.0045 (15)	0.0078 (15)	0.0148 (14)
O14	0.0424 (11)	0.0503 (11)	0.0346 (10)	0.0144 (9)	−0.0010 (8)	−0.0037 (8)
C15	0.0469 (16)	0.0475 (16)	0.0432 (15)	0.0129 (14)	−0.0060 (13)	−0.0047 (13)
C16	0.0356 (14)	0.0421 (15)	0.0401 (14)	0.0084 (12)	−0.0039 (12)	−0.0004 (12)
C17	0.0545 (19)	0.0481 (17)	0.0533 (18)	−0.0031 (15)	−0.0016 (15)	0.0022 (14)
C18	0.0516 (19)	0.082 (3)	0.0494 (19)	−0.0063 (18)	0.0107 (15)	0.0127 (17)
C19	0.0538 (19)	0.078 (2)	0.0425 (17)	0.0188 (18)	0.0036 (15)	−0.0082 (16)
C20	0.0566 (19)	0.0456 (17)	0.0473 (17)	0.0131 (15)	−0.0035 (15)	−0.0065 (13)
C21	0.0411 (16)	0.0430 (15)	0.0432 (15)	0.0047 (13)	0.0000 (12)	0.0006 (12)
S22	0.0295 (3)	0.0466 (4)	0.0330 (3)	0.0008 (3)	0.0022 (3)	−0.0022 (3)
O23	0.0300 (9)	0.0565 (12)	0.0401 (10)	0.0032 (9)	−0.0036 (8)	−0.0038 (9)
O24	0.0428 (11)	0.0533 (12)	0.0379 (10)	−0.0057 (9)	0.0086 (9)	−0.0006 (9)
C26	0.0448 (16)	0.0470 (16)	0.0374 (14)	−0.0002 (13)	0.0059 (12)	0.0012 (12)
C27	0.0576 (19)	0.0493 (17)	0.0380 (16)	0.0012 (15)	0.0049 (14)	−0.0051 (13)
C28	0.0494 (17)	0.0473 (16)	0.0461 (16)	−0.0006 (14)	−0.0014 (14)	−0.0003 (13)
C31	0.084 (3)	0.0484 (19)	0.065 (2)	−0.0099 (18)	0.003 (2)	−0.0034 (16)
C29	0.0523 (19)	0.0571 (19)	0.0443 (17)	−0.0119 (16)	0.0052 (14)	0.0047 (14)
C30	0.0468 (17)	0.0580 (18)	0.0354 (14)	−0.0075 (14)	0.0084 (13)	−0.0043 (13)
C25	0.0313 (13)	0.0460 (15)	0.0347 (13)	0.0014 (12)	0.0012 (11)	−0.0004 (12)

### Geometric parameters (Å, º)

**Table:**

C1—O12	1.419 (4)	C15—C16	1.500 (4)
C1—C2	1.514 (4)	C16—C17	1.381 (4)
C1—C11	1.519 (4)	C16—C21	1.386 (4)
C2—N3	1.483 (3)	C17—C18	1.384 (5)
C2—C13	1.554 (4)	C18—C19	1.379 (5)
N3—C4	1.480 (3)	C19—C20	1.368 (5)
N3—S22	1.622 (2)	C20—C21	1.380 (4)
C4—C5	1.509 (4)	S22—O24	1.438 (2)
C5—C6	1.518 (4)	S22—O23	1.441 (2)
C6—C7	1.377 (4)	S22—C25	1.765 (3)
C6—C11	1.413 (4)	C26—C25	1.382 (4)
C7—C8	1.397 (4)	C26—C27	1.390 (4)
C8—O14	1.369 (3)	C27—C28	1.381 (4)
C8—C9	1.385 (4)	C28—C29	1.396 (4)
C9—C10	1.384 (4)	C28—C31	1.506 (4)
C10—C11	1.377 (4)	C29—C30	1.373 (4)
O14—C15	1.443 (3)	C30—C25	1.386 (4)
			
O12—C1—C2	106.2 (2)	C17—C16—C21	119.2 (3)
O12—C1—C11	113.4 (2)	C17—C16—C15	120.8 (3)
C2—C1—C11	116.3 (3)	C21—C16—C15	120.0 (3)
N3—C2—C1	112.2 (2)	C16—C17—C18	120.0 (3)
N3—C2—C13	111.5 (2)	C19—C18—C17	120.2 (3)
C1—C2—C13	111.6 (3)	C20—C19—C18	120.0 (3)
C4—N3—C2	119.5 (2)	C19—C20—C21	120.1 (3)
C4—N3—S22	121.29 (18)	C20—C21—C16	120.5 (3)
C2—N3—S22	115.37 (17)	O24—S22—O23	117.62 (12)
N3—C4—C5	113.2 (2)	O24—S22—N3	110.87 (12)
C4—C5—C6	114.3 (2)	O23—S22—N3	107.28 (11)
C7—C6—C11	119.1 (3)	O24—S22—C25	106.78 (12)
C7—C6—C5	119.9 (2)	O23—S22—C25	107.70 (12)
C11—C6—C5	121.1 (2)	N3—S22—C25	105.96 (13)
C6—C7—C8	121.5 (2)	C25—C26—C27	118.9 (3)
O14—C8—C9	124.7 (2)	C28—C27—C26	121.3 (3)
O14—C8—C7	115.8 (2)	C27—C28—C29	118.3 (3)
C9—C8—C7	119.5 (2)	C27—C28—C31	121.0 (3)
C10—C9—C8	118.6 (3)	C29—C28—C31	120.6 (3)
C11—C10—C9	122.9 (3)	C30—C29—C28	121.3 (3)
C10—C11—C6	118.3 (3)	C29—C30—C25	119.3 (3)
C10—C11—C1	119.0 (3)	C26—C25—C30	120.8 (3)
C6—C11—C1	122.6 (3)	C26—C25—S22	119.8 (2)
C8—O14—C15	116.0 (2)	C30—C25—S22	119.2 (2)
O14—C15—C16	108.8 (2)		
			
O12—C1—C2—N3	−53.8 (3)	O14—C15—C16—C17	−80.1 (3)
C11—C1—C2—N3	73.3 (3)	O14—C15—C16—C21	102.7 (3)
O12—C1—C2—C13	−179.8 (2)	C21—C16—C17—C18	0.0 (5)
C11—C1—C2—C13	−52.6 (3)	C15—C16—C17—C18	−177.2 (3)
C1—C2—N3—C4	−64.1 (3)	C16—C17—C18—C19	0.0 (5)
C13—C2—N3—C4	61.9 (3)	C17—C18—C19—C20	−0.2 (5)
C1—C2—N3—S22	137.5 (2)	C18—C19—C20—C21	0.3 (5)
C13—C2—N3—S22	−96.4 (3)	C19—C20—C21—C16	−0.3 (5)
C2—N3—C4—C5	69.7 (3)	C17—C16—C21—C20	0.1 (4)
S22—N3—C4—C5	−133.3 (2)	C15—C16—C21—C20	177.4 (3)
N3—C4—C5—C6	−79.2 (3)	C4—N3—S22—O24	−114.3 (2)
C4—C5—C6—C7	−123.1 (3)	C2—N3—S22—O24	43.7 (2)
C4—C5—C6—C11	56.9 (4)	C4—N3—S22—O23	15.4 (3)
C11—C6—C7—C8	1.3 (4)	C2—N3—S22—O23	173.3 (2)
C5—C6—C7—C8	−178.6 (3)	C4—N3—S22—C25	130.2 (2)
C6—C7—C8—O14	176.6 (3)	C2—N3—S22—C25	−71.8 (2)
C6—C7—C8—C9	−4.1 (4)	C25—C26—C27—C28	0.7 (5)
O14—C8—C9—C10	−177.5 (3)	C26—C27—C28—C29	0.8 (5)
C7—C8—C9—C10	3.2 (4)	C26—C27—C28—C31	−179.1 (3)
C8—C9—C10—C11	0.3 (5)	C27—C28—C29—C30	−0.8 (5)
C9—C10—C11—C6	−3.1 (5)	C31—C28—C29—C30	179.1 (3)
C9—C10—C11—C1	173.4 (3)	C28—C29—C30—C25	−0.8 (5)
C7—C6—C11—C10	2.2 (4)	C27—C26—C25—C30	−2.3 (4)
C5—C6—C11—C10	−177.9 (3)	C27—C26—C25—S22	172.5 (2)
C7—C6—C11—C1	−174.1 (3)	C29—C30—C25—C26	2.4 (5)
C5—C6—C11—C1	5.8 (5)	C29—C30—C25—S22	−172.4 (2)
O12—C1—C11—C10	−116.8 (3)	O24—S22—C25—C26	7.2 (3)
C2—C1—C11—C10	119.7 (3)	O23—S22—C25—C26	−120.0 (2)
O12—C1—C11—C6	59.5 (4)	N3—S22—C25—C26	125.5 (2)
C2—C1—C11—C6	−64.0 (4)	O24—S22—C25—C30	−177.9 (2)
C9—C8—O14—C15	1.8 (4)	O23—S22—C25—C30	54.9 (3)
C7—C8—O14—C15	−178.9 (2)	N3—S22—C25—C30	−59.7 (3)
C8—O14—C15—C16	175.5 (2)		

### Hydrogen-bond geometry (Å, º)

Cg1, Cg2 and Cg3 are the centroids of rings C6–C11, C16–C21 and C25–C30, respectively.

**Table:**

*D*—H···*A*	*D*—H	H···*A*	*D*···*A*	*D*—H···*A*
O12—H12···O23^i^	0.83	2.22	3.034 (3)	169
C2—H2···O24^ii^	0.99	2.52	3.265 (3)	132
C18—H18···*Cg*3^iii^	0.94	2.89	3.738 (4)	150
C20—H20···*Cg*1^iv^	0.94	2.83	3.631 (3)	144
C29—H29···*Cg*2^v^	0.94	2.76	3.545 (3)	142

Symmetry codes: (i) *x*−1, *y*, *z*; (ii) *x*−1/2, *y*, −*z*+1/2; (iii) *x*−3/2, −*y*+1/2, −*z*+1; (iv) −*x*−1, −*y*+1, −*z*+1; (v) *x*+1/2, −*y*+1/2, −*z*+1.
